# Changes in plasticity of the pelvic girdle from infancy to late adulthood in *Homo sapiens*

**DOI:** 10.1038/s41598-023-36703-2

**Published:** 2023-06-15

**Authors:** Anna Maria Kubicka

**Affiliations:** 1 Department of Zoology, Poznań University of Life Sciences, Wojska Polskiego 71C, 60-625 Poznań, Poland; 2 PaleoFED Team, Département Homme et Environnement, Muséum National d’Histoire Naturelle. Musée de l’Homme, UMR 7194, CNRS, Place du Trocadéro 17, 75016 Paris, France

**Keywords:** Anthropology, Biological anthropology

## Abstract

Previous research on the effects of body mass on the pelvic girdle focused mostly on adult females and males. Because the ontogenetic plasticity level in the pelvis remains largely unknown, this study investigated how the association between body mass index (BMI) and pelvic shape changes during development. It also assessed how the large variation in pelvic shape could be explained by the number of live births in females. Data included CT scans of 308 humans from infancy to late adulthood with known age, sex, body mass, body stature, and the number of live births (for adult females). 3D reconstruction and geometric morphometrics was used to analyze pelvic shape. Multivariate regression showed a significant association between BMI and pelvic shape in young females and old males. The association between the number of live births and pelvic shape in females was not significant. Less plasticity in pelvic shape in adult females than during puberty, perhaps reflects adaptation to support the abdominopelvic organs and the fetus during pregnancy. Non-significant susceptibility to BMI in young males may reflect bone maturation accelerated by excessive body mass. Hormonal secretion and biomechanical loading associated with pregnancy may not have a long-term effect on the pelvic morphology of females.

## Introduction

Shape variation in the human pelvis was analyzed in the context of sexual dimorphism^1^, age^2^, thermoregulation^3^, past population history^4^, mechanical constraints^5–7^, stature^8^, evolution, and birth canal variation^3,9,10^. Much attention was also paid to pelvic morphology in females, which reflects competing functional demands (e.g. locomotor, childbirth, abdominopelvic organ support)^11–13^. Huseynoy et al.^11^ showed that the female pelvis is characterized by great plasticity in the context of obstetrical dimensions during ontogeny. Developmental trajectories of females and males separate in early infancy and change substantially with increasing obstetrical dimensions in females until age 40–45 years when the female pelvis begins to change in parallel with the male trajectory^11^. The greatest differences in pelvic shape between the sexes are achieved at the time of maximum fertility in females^11^. The amount and duration of hormone secretion (i.e. estrogen and androgen) are likely the main drivers of these differences in developmental trajectories between sexes by inducing pelvic bone remodeling^14,15^. The pattern of human pelvic dimorphism is conservative compared to the highly variable magnitude of the sex differences^15^, which may depend partly on environmental factors such as mechanical loadings. However, the potential influence of bone-loading conditions such as body mass on the developmental trajectories of pelvises in females and males remains relatively unexplored.

While various studies have investigated the effects of body mass and posture on the human pelvis, they focused on mature females^16^ or males^17^. One longitudinal study^18^ showed that adolescents with higher body fat tended to have a larger pelvic width, possibly due to adipose estrogenization. However, a higher biomechanical load could not be excluded as a causal factor^18^. Nevertheless, the association between body mass and pelvic shape during growth has yet to be explored. The human pelvis is one of the skeleton’s most important weight-bearing bone structures due to its bipedal locomotion. Moreover, it provides attachments for muscles, supports and protects the abdominal and reproductive organs, stabilizes the spine, and in females, enables childbirth^19–22^. Therefore, it can be assumed that mechanical loading, such as body mass, is one of the factors inducing the pelvic girdle’s developmental trajectories through adaptive bone remodeling. Moreover, considering the obstetrical requirements of females and variable ontogenetic plasticity of the pelvis^11^, the morphological response of pelvic shape can differ in magnitude between sexes depending on the ontogenetic phase.

Another question is whether the number of live births can shape the functional plasticity of the pelvic girdle in females. For example, a mouse model showed that females with the largest number of offspring had the most divergent pelvic shape^23^. However, previous human studies found no differences in pelvic shape between parous and nonparous females^11,24^. Despite that, each pregnancy and parturition increases pelvic organ (urethral and bladder) mobility^25^ and is associated with prolonged hormonal secretion (e.g. high levels of estrogen, progesterone, and relaxin) that stimulates bone remodeling and weakens pelvic ligaments^26,27^, which can lead to pelvic floor dysfunction^28–30^. Therefore, does prolonged hormonal stimulation and biomechanical stress associated with each subsequent pregnancy increase adaptation in obstetrical dimensions in females? Consequently, the potential relationship between the number of live births and pelvic shape in females at the age of the greatest fertility requires reassessment.

In summary, this study: (1) assesses the relationship between pelvic shape and body mass category of females and males during ontogeny; (2) examines the relationship between the number of live births and the pelvic shape in females at the age of greatest fertility (i.e. 25–45 years)^11,31^. It is expected that female pelvis shape be more influenced by body mass than the male pelvis, as females exhibit higher ontogenetic plasticity (Hypothesis 1). Secondly, the pelvic shape is expected to change with the number of live births in females of reproductive age, given that the obstetrical dimensions of the female pelvis are induced by prolonged hormonal stimulation during pregnancy (Hypothesis 2). To test these two hypotheses, a three-dimensional (3D) reconstruction of the pelvic girdle was integrated with geometric morphometrics to assess pelvic plasticity from infancy to late adulthood in a large forensic sample of 308 individuals.

## Results

**Figure 1:**
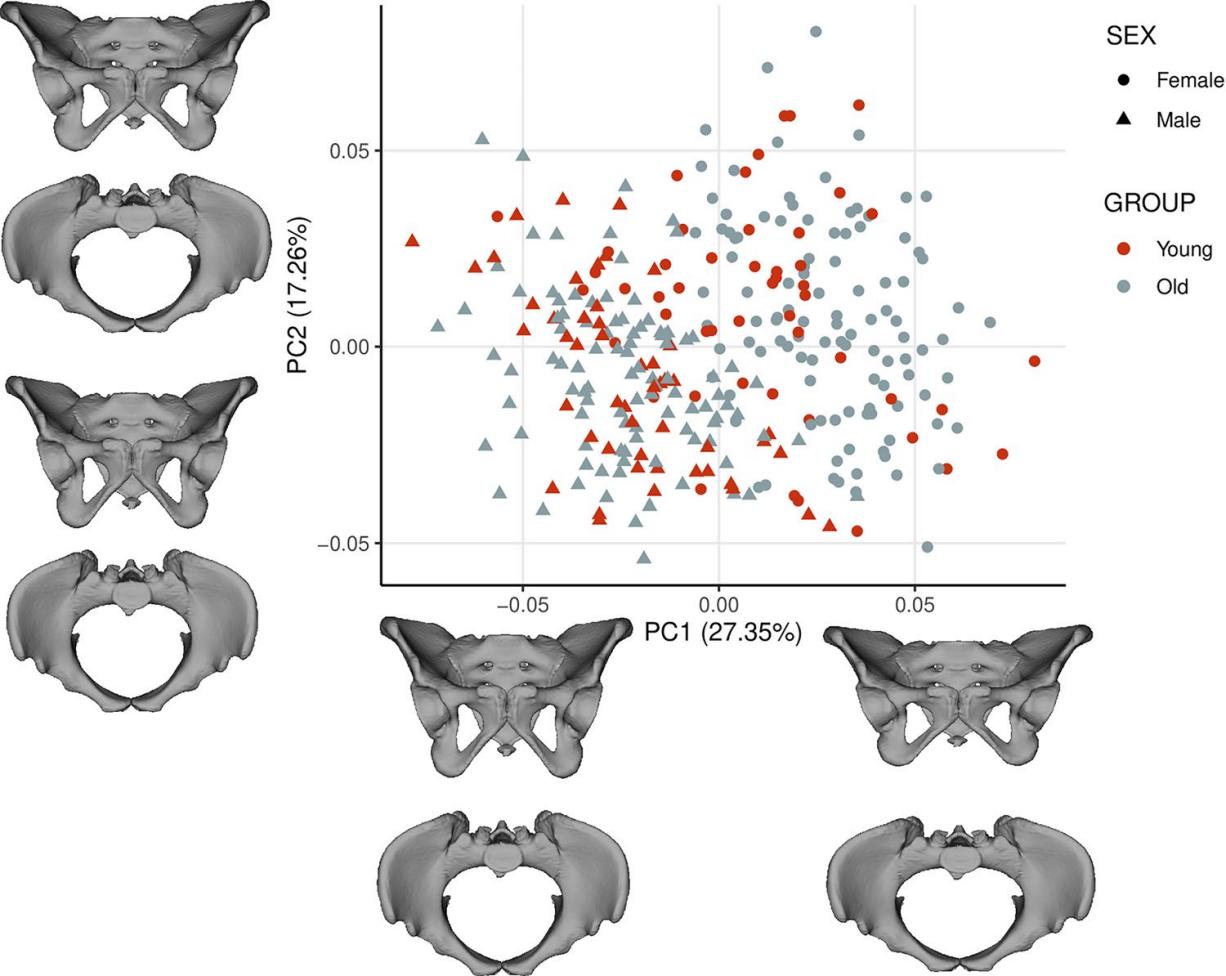
PCA on the multivariate residuals.

**Figure 2:**
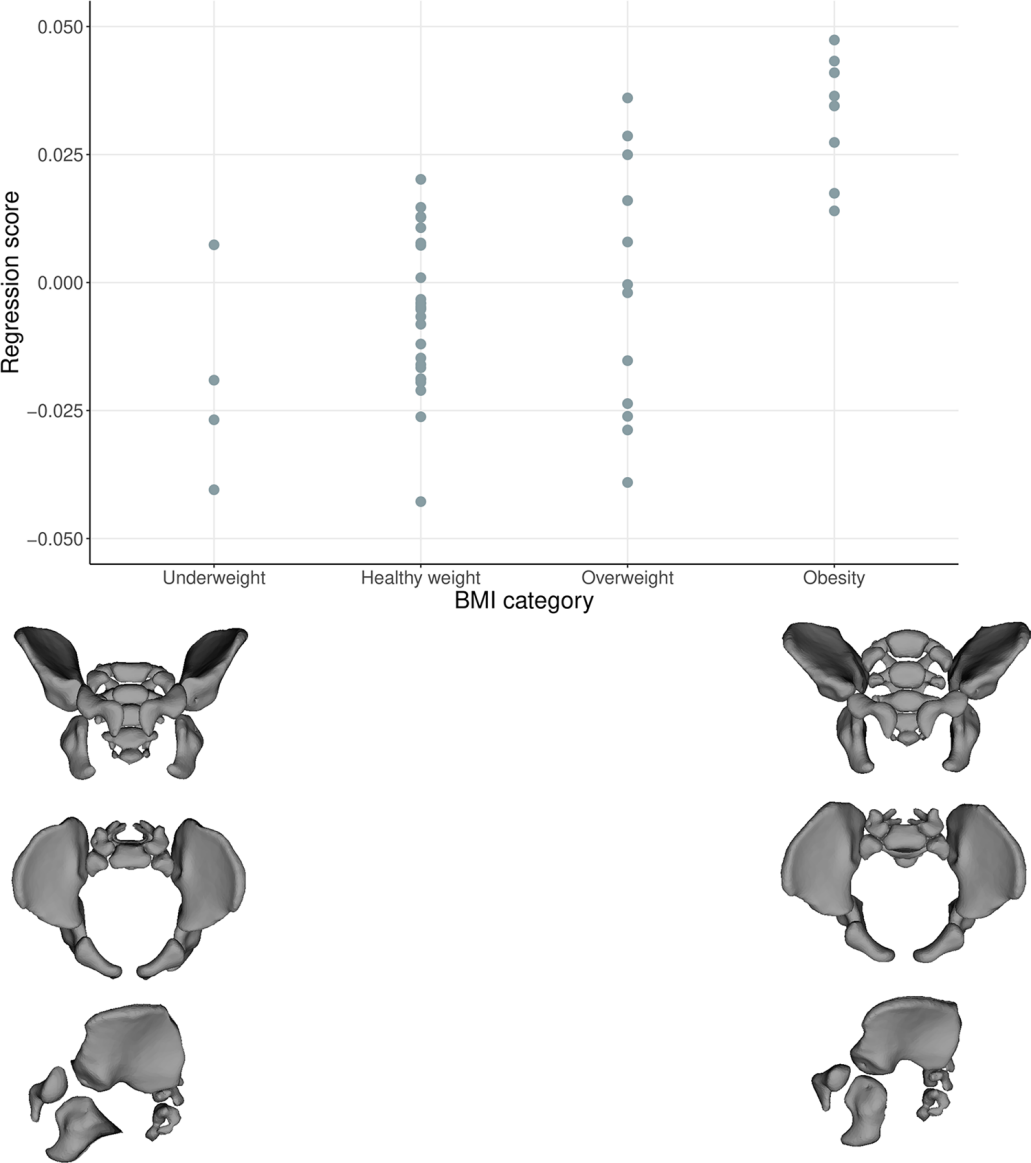
The relationship between BMI category and pelvic shape (Procrustes coordinates) in females from the young group (0–25 years).

**Figure 3:**
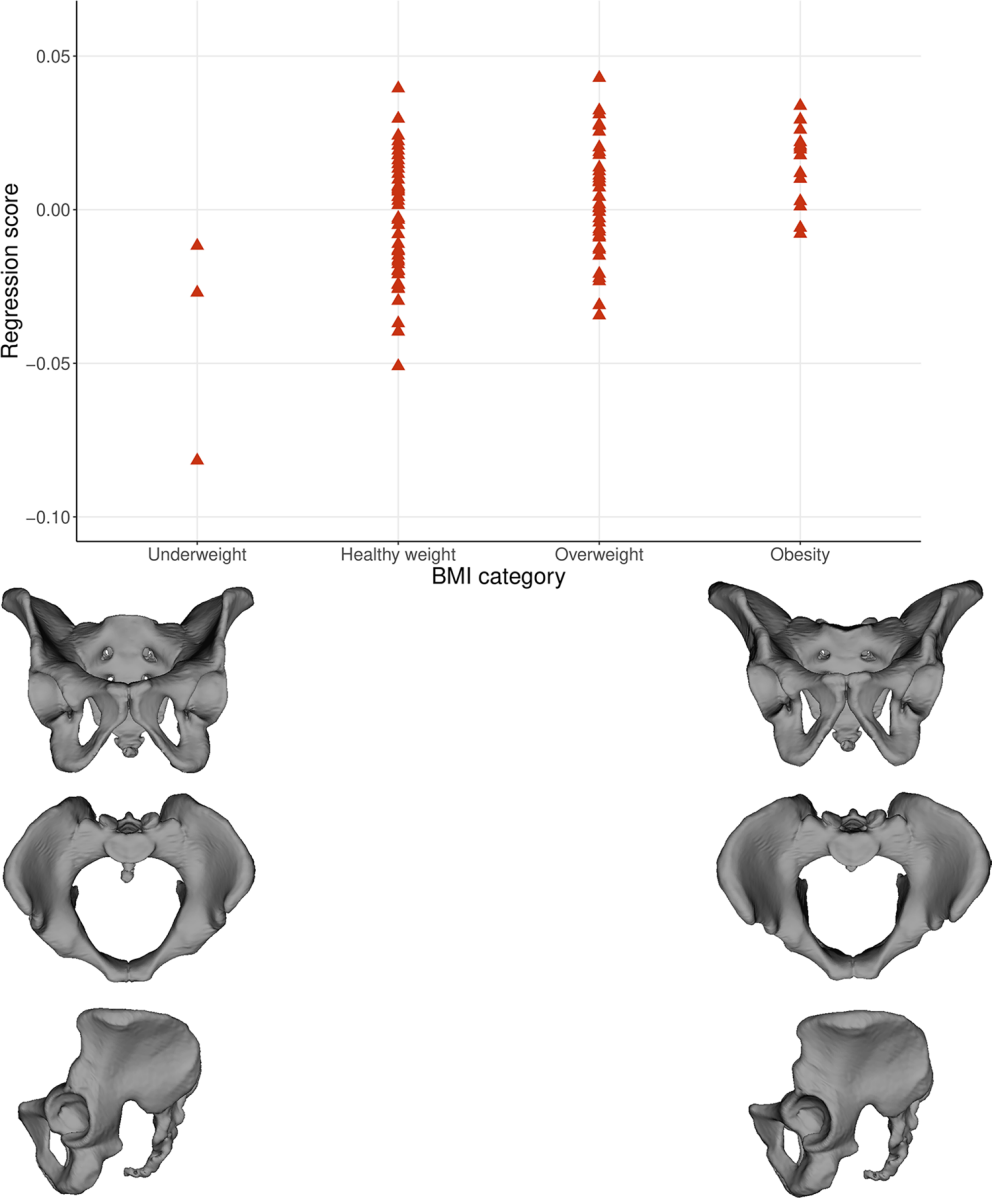
The relationship between BMI category and pelvic shape (Procrustes coordinates) in males from the old group (> 25 years). The warped surface of the pelvic girdle represents a 27-year-old male.

PCA was performed on the multivariate regression residuals to visualize the variation of the complete data set without ontogenetic allometry. Females and males from both groups (young and old) are characterized by relatively similar shape variation, which minimizes the likelihood of a negative impact on further regression results. In addition, Fig. 1 shows that the age groups of both sexes are not differentiated along PC1 and PC2. PC1 and PC2 explained 27.35% and 17.26% of the total variation in the data set, respectively. Both sexes diverged along the PC1 axis representing the ilium’s bending and widening and the size of the subpubic angle (Movies 1S, 2S). PC2 represents the width of the pelvic inlet and bending of the ischium (Fig. 1, Movies 3S, 4S). The multivariate regression of pelvic shape and BMI categories was significant in females and males from the young (0–25 years) and old (> 25 years) groups (Table 1; Figs. 2, 3). The pelvic girdle of young females with underweight and healthy BMIs (N = 30) showed relatively more upright ilium and prominent ischial spines and relatively higher everted sacrum than those with overweight and obese BMIs (N = 20; Fig. 2; for additional visualization in three orientations, see Movies 5S, 6S and 7S). However, the relative anteroposterior and transverse dimensions of the pelvic outlet and inlet did not seem to differ regarding BMI categories in young females (Fig. 2; Movies 5S, 6S and 7S). In males with underweight and healthy BMIs (N = 51) in the old group (> 25 years), the pelvic girdle had a relatively: more upright ilium, a higher sacrum base, a larger subpubic angle, a more forward acetabulum, more backward spina iliaca anterior-inferior, and anterior–superior and less triangular pelvic outlet than those with overweight and obese BMIs (N = 50; Fig. 3; Movies 8S, 9S and 10S). The association between the pelvic shape and the number of live births in 43 females aged 25–45 was insignificant (predicted 1.72% of shape variation; p = 0.715; Fig. 3S).

**Table 1:** The multivariate regression between pelvic shape and the BMI categories in the young and old groups by sex.

Sample	Female			Male		
	N	Predicted [%]	*P*	N	Predicted [%]	*P*
Young group (0–25)	50	5.48%	**0.008**	50	2.80%	0.166
Old group (> 25)	107	1.28%	0.178	101	2.61%	**0.004**

*N* number of individuals in the group, *Predicted %* percent of the predicted shape, *P* p value of the permutation test with 10,000 randomization rounds, bold indicates statistically significant p value at level < 0.05.

## Discussion

This is the first study investigating the association between body mass and pelvic shape changes during development. The geometric morphometric analysis showed a significant relationship between pelvic shape and BMI category only in young females (0–25 years) and old males (> 25 years). Therefore, these results indicate that before the end of maturation (i.e. 0–25 years), the female pelvis shows greater plasticity and susceptibility to body mass than the pelvic girdle of similarly aged males. However, is this greater plasticity during female development an adaptation to future obstetrical challenges?

Comparing the obtained pattern in pelvis shape variability during growth to other studies is difficult since they focused mainly on adults^16,17,32–34^. Nevertheless, the observed differences in pelvic shape between BMI categories result from a highly integrated system in which functional and anatomical components interact^35^. However, a previous study showed that not all pelvic components exhibit the same magnitude of interaction^2^. The linear dimensions and correlation matrices showed that the ilium was less constrained than the pubis in both sexes and than the pelvis outlet and inlet in females^2^. These findings may explain why this geometric morphometric analysis showed more pronounced changes in ilium orientation than in pelvic canal shape in young females. The birth canal shape in young females likely shows a higher canalization and is under stronger evolutionary constraints than non-obstetrical traits that respond independently. Therefore, the results suggest that body mass does not determine pelvis inlet and outlet shape during development, although it may influence variability in canal dimensions in adult females^32^. In addition, the widening of the ilium in young females may be a biomechanical response to the mechanical loading of excess body mass.

The BMI categories were equally numerous in both sexes. In addition, both sexes in the young and old groups are characterized by relatively similar shape variation. Therefore, the absence of pelvic plasticity with BMI in young males is not due to sampling bias or differences in the variation between groups. These results might reflect differences in the ontogenetic pelvic shape trajectories between sexes^36^ and sex-specific hormone susceptibility. Obese boys and girls show accelerated bone maturation and earlier pubertal events partially due to increased leptin and irisin concentration^37,38^. Being overweight or obese might accelerate the already faster shape maturation rate of the pelvic girdle, especially the ilium, in young males, making it less prone to shape changes. Sex hormones that play a role in leptin synthesis may enhance this effect in young boys^38^. However, it must be emphasized that the complex relationship between BMI and sex hormones leptin and irisin in subadults remains largely unknown. Therefore, further studies are needed to confirm this hypothesis.

Kurki^39^ and Huseynov et al.^11^ found that the pelvic dimensions of adult females and males are equally plastic. Shorter and lower-weight females have relatively large inter-landmark birth canal distances^16^. Similarly, body mass is the main factor explaining pelvic shape variability in modern and medieval males^17^. These patterns contrast with the results of this study, where the relationship between BMI category and pelvic shape was significant only for males in the old group (> 25 years). The geometric morphometric analysis showed changes in the ilium, subpubic angle size, and acetabular orientation of the male pelvis. One potential explanation for these discrepancies is that the pelvis of modern adult females is less plastic and differs from that of Ricklan et al.^16^. Because the human pelvis has a neutral variance pattern^34^, discrepancies among studies can reflect differences in population histories of their investigated groups. Another potential cause is differences in their methodological approaches. Ricklan et al.^16^ examined pelvic dimensions, body mass, and height, while this study explored BMI and shape using geometric morphometrics. Nevertheless, BMI categories did not explain shape variation in the female pelvis, even though the pelvic girdle is susceptible to changes after puberty^11^. The absence of shape changes due to excess body mass is potentially an advantage in heavier adult females since the pelvis supports the abdominopelvic organs and fetus during pregnancy^19^.

The geometric morphometric analysis found that the number of live births could not explain shape variation in the female pelvis. Previous studies on females with known maternal status provided similar results since the pelvis of parous and nonporous females did not differ significantly^11^. Therefore, increasing mobility of the pubic symphysis and sacroiliac joints during pregnancy^40,41^ may not majorly affect pelvic morphology. Nevertheless, this finding is important in the context of the secular trend in maternal obesity, especially in low- and middle-income countries^42^. Wells^42^ found that overweight and obese mothers had higher risks of macrosomia in offspring and obstructed labor. The absence of changes in the pelvic shape, particularly obstetrical traits, in overweight and obese females, resulting in a birth canal ill-suited to large infants, can explain the disproportional cephalopelvic risk.

The relationship between pelvic capacity and body size is population-specific due to variation in body proportions^43,44^. Moreover, there are interpopulation differences in postnatal ontogeny trajectories of pelvic shape^36^. Because this study examined a relatively homogenous group born in the United States (details on ethnicity in Table 2S), albeit from different states, the pelvic plasticity patterns observed cannot be considered universal. Studies on other populations are needed to clarify whether body mass determines the developmental processes leading to the mature pelvis in females and males. Other aspects can also advance understanding of the functional plasticity of the human pelvis. For example, since the secular trend of pubertal timing is observed worldwide^45^, it is important to study whether earlier maturation influences the functional plasticity of the pelvic girdle, which could be investigated by menarcheal age since it is the most accurate pubertal stage in females^46^. Demographic and medical data of modern populations would be invaluable in this regard. Other studies^16,17^ have provided invaluable information on pelvic morphological variation. However, their findings cannot be used as a general pattern since they separately focused on mature males and females. Therefore, studies should focus on both sexes at different ontogenetic phases since this study showed different plasticities in males and females during development. While most studies on the human pelvis are observational^47^, it is impossible to investigate cause-effect relationships. Therefore, large-scale forensic data with demographic and medical information may be useful in future experimental studies and animal models.

## Material and methods

The material consisted of high-resolution full-body CT scans of 308 humans (157 females and 151 males) from infancy to late adulthood (Tables 1S and 2S) with information on sex, age in months at death, living weight, and height. The number of live births of 43 females aged 25–45 was also collected. The volumetric, demographic, and medical data were acquired from the New Mexico Decedent Image Database^48^ (details in the Supplement). Next, body mass index (BMI) was calculated for each individual using the equation: BMI = body mass (kg)/body height (m^2^). In individuals over 20 years of age, BMI was classified based on the following ranges: underweight (≤ 18.5), healthy weight (18.5–24.9), overweight (25.0–29.9), and obese (≥ 30.0). Individuals under 20 years of age were classified using sex-specific growth charts as underweight (< 10th percentile), healthy weight (10–85th), overweight (85–95th), and obese (> 95th).

Whole body CT scans were acquired using a standard protocol with 0.5 mm slice thickness. 3D reconstruction and mesh cleaning of the pelvic girdle was performed using free software such as 3D Slicer (v.4.11)^49^ and Gom Inspect (v. Hotfix 4)^50^, respectively. One pelvis on which 61 landmarks (LM) were manually digitized was selected as a template (Table 3S). Next, 476 surface semilandmarks (SLM) were automatically generated from the template mesh. Then, SLM sliding was performed based on the manually digitized 61 LM on each pelvic girdle using the minimum bending energy criterion of the SlicerMorph extension in 3D Slicer (details in the Supplement). The LM set contained 10 LM pairs that fuse during pelvic development. Therefore, in individuals with completed fusion, the mean position was calculated for each of these 10 LM pairs (Figs. 1S, 2S).

The raw coordinate data were divided into two groups, young (individuals aged 0–25) and old (individuals aged > 25), to separately analyze changes in the pelvic shape during stages of intense and complete skeletal development, respectively. A Generalized Procrustes Analysis was performed to remove the differences in location, orientation, and scale of the raw coordinates and to obtain the Procrustes coordinates (details in the Supplement). Multivariate regression with the permutation test (10,000 rounds) was performed between the Procrustes coordinates and the centroid size in young and old groups to test for ontogenetic allometry. The results were statistically significant in both groups (Table 4S). Therefore, all further geometric morphometric analysis was performed on the multivariate regression residuals to remove the ontogenetic allometry effect. This approach was because size can be a crucial component of the shape variation during growth^51^, and the study analyses individuals at different developmental stages. Therefore, removing the allometry effect ensures that the detected variation in the pelvic shape was not due to differences in the geometric size. This was especially important in the young group, which, due to the rapid pace of development, is characterized by high variation in size.

Principal component analysis (PCA) on the multivariate regression residuals was used to visualize the variation pattern of the pelvic shape. The PCA was carried out without allometric effect to explore whether both groups (young and old) within sex show relatively similar shape variation, which is important for the potential impact on the results of further geometric morphometric analysis (i.e. the multivariate regression).

Two further geometric morphometric analyses were also carried out without an allometric effect. The association between pelvic shape (i.e., the residuals of the allometric regression) and BMI categories by sex was performed using the multivariate regression separately in the young and old groups. The multivariate regression was also used to investigate the association between pelvic shape (i.e. the residuals of the allometric regression) and the number of live births in 43 females aged 25–45 (Table 6S). This geometric morphometric analysis focused on females in a narrow age range since the female pelvis shows relevant obstetrical dimensions during this period^11^. Statistical analyses were performed using MorphoJ (v.1.07a). In turn, the R software (v.4.2.0) with the package ggplot2^52^ was used to create the figures. All results with p < 0.05 were considered statistically significant.

The author confirms that all stages of the research (collection, CT scanning, and analysis) were performed in accordance with the fundamental ethical principles and regulations on the analysis of human remains. All analysed CT scans were derived from the New Mexico Decedent Image Database and no new patients were scanned for this study. The acquisition of the medical image series and biological data of deceased persons was done retrospectively in compliance with the Declaration of Helsinki for the protection of data privacy. CT scans of decedents are not regarded as human subjects under U.S. federal laws pertaining to research on human subjects. Further, Health Insurance Portability and Accountability Act (HIPAA) protections do not apply to data obtained in the investigation of a person's death. Moreover, the scans do not comprise any personal identifiable information, and because the next of kin interviewed were not subjects of research, no review was required by the Institutional Review Board (IRB) of the New Mexico Decedent Image Database. However, the project descriptions and research design were reviewed by the College of Arts and Sciences, Department of Anthropology who considered the research to collect the derivatives was exempt from IRB review.

## Supplementary Information

Supplementary Video 1.

Supplementary Video 2.

Supplementary Video 3.

Supplementary Video 4.

Supplementary Video 5.

Supplementary Video 6.

Supplementary Video 7.

Supplementary Video 8.

Supplementary Video 9.

Supplementary Video 10.

Supplementary Information.

## Data Availability

The data that support the findings of this study are available from the New Mexico Decedent Image Database but restrictions apply to the availability of these data, which were used under license for the current study, and so are not publicly available. Data are however available from the authors upon reasonable request and with permission of the New Mexico Decedent Image Database. To request the data, please visit the website: https://nmdid.unm.edu/ and read the information for researchers in the tab “How to use” or contact directly with the New Mexico Decedent Image Database via the project email address (NMDID@unm.edu). 3D raw coordinates with biological variables, R code used to create figures, PCA coefficients and morphoj file are available on an external repository (Mendeley Data: https://doi.org/10.17632/k85nv2j2p5.1)^53^.
