# Supplementary material for: Changes in plasticity of the pelvic girdle from infancy to late adulthood in Homo sapiens
Source: Sci Rep. 2023 Jun 15;13:9698. doi: 10.1038/s41598-023-36703-2 (PMC10272276; doi:10.1038/s41598-023-36703-2)
Supplement: Supplementary file 11 — Supplementary Information. [file 41598_2023_36703_MOESM11_ESM.docx]

**Supplement**

**Material and Methods**

The volumetric and demographic data were obtained from the New Mexico Decedent Image Database, which includes whole-body CT scans of over 15,000 New Mexicans who died between 2010–2017^1^. The growth charts used with the young group (<20 years) were developed by the National Center for Health Statistics in collaboration with the National Center for Chronic Disease Prevention and Health Promotion. The clinical sex-based growth charts (3^rd^–97^th^ percentile) for infant length-by-age and weight-by-age were used for individuals from birth to 36 months. The body mass of individuals aged 36 months to 20 years was categorized based on BMI-for-age clinical growth charts (3^rd^–97^th^ percentile) for children and adolescents.

Table 1S Sample size by age, sex, and BMI category

| Age category  [years] | Female | | Male | |  |
| --- | --- | --- | --- | --- | --- |
|  | Total | BMI category [N] | Total | BMI category [N] | |
| 0-5 | 10 | UN [1], HW [4], OV[3], OB[2] | 10 | UN [1], HW [4], OV[2], OB[3] | |
| 5-10 | 10 | UN [0], HW [3], OV[2], OB[5] | 10 | UN [1], HW [5], OV[2], OB[2] | |
| 10-15 | 10 | UN [0], HW [5], OV[5], OB[0] | 10 | UN [0], HW [1], OV[6], OB[3] | |
| 15-20 | 10 | UN [1], HW [7], OV[1], OB[1] | 10 | UN [1], HW [7], OV[2], OB[0] | |
| 20-25 | 10 | UN [2], HW [7], OV[1], OB[0] | 10 | UN [0], HW [7], OV[3], OB[0] | |
| 25-30 | 10 | UN [0], HW [5], OV[4], OB[1] | 10 | UN [0], HW [5], OV[2], OB[3] | |
| 30-35 | 12 | UN [0], HW [6], OV[5], OB[1] | 10 | UN [0], HW [4], OV[3], OB[3] | |
| 35-40 | 11 | UN [0], HW [7], OV[4], OB[0] | 11 | UN [0], HW [4], OV[7], OB[0] | |
| 40-45 | 10 | UN [0], HW [8], OV[1], OB[1] | 10 | UN [0], HW [7], OV[3], OB[0] | |
| 45-50 | 11 | UN [0], HW [6], OV[4], OB[1] | 10 | UN [1], HW [5], OV[2], OB[2] | |
| 50-55 | 10 | UN [0], HW [4], OV[5], OB[1] | 9 | UN [0], HW [4], OV[5], OB[0] | |
| 55-60 | 10 | UN [3], HW [6], OV[0], OB[1] | 10 | UN [1], HW [6], OV[3], OB[0] | |
| 60-65 | 10 | UN [2], HW [7], OV[1], OB[0] | 10 | UN [0], HW [4], OV[3], OB[3] | |
| 65-70 | 10 | UN [0], HW [4], OV[5], OB[1] | 9 | UN [1], HW [4], OV[4], OB[0] | |
| 70-75 | 11 | UN [1], HW [9], OV[1], OB[0] | 10 | UN [0], HW [4], OV[3], OB[3] | |
| 75-80 | 2 | UN [0], HW [1], OV[1], OB[0] | 2 | UN [0], HW [1], OV[1], OB[0] | |
| Sum | 157 | UN [10], HW [89], OV[43], OB[15] | 151 | UN [6], HW [72], OV[51], OB[22] | |

Total – number of individuals in the age category, N – number of individuals classified to the BMI category, UN – underweight, HW – healthy weight, OV – overweight, OB - obesity

The study material is a relatively homogenous group, as all individuals were born in the United States. The New Mexico Decedent Image Database also contains information on ethnicity collected for each individual (Table 2S).

Table 2S. Sample size by sex and ethnicity

| Sample | Female | | Male | |
| --- | --- | --- | --- | --- |
|  | Hispanic or Latino | Not Hispanic, Latino  or Middle Eastern | Hispanic or Latino | Not Hispanic, Latino  or Middle Eastern |
| Young group (<25) | 21 | 29 | 15 | 35 |
| Old group (>25) | 24 | 83 | 14 | 87 |
| Sum | 45 | 112 | 29 | 122 |

The locations of the 61 LMs on the pelvic girdle were selected based on previous studies^2,3^. Table 3S and Figure 1S show the definition and visualization of the LM distribution, respectively.

Table 3S. LM location definition

| LM | Description |
| --- | --- |
| 1-2 | Pubic symphysis: most superior-anterior point |
| 3-4 | Pubic symphysis: most superior-posterior point |
| **5-6** | Ischiopubic juncture: anterior fusion point on the pubic bone |
| **7-8** | Ischiopubic juncture: anterior fusion point on the ischium bone |
| **9-10** | Ischiopubic juncture: posterior fusion point on the pubic bone |
| **11-12** | Ischiopubic juncture: posterior fusion point on the ischium bone |
| 13-14 | Foramen obturatum: most inferior point |
| 15-16 | Corpus ossis ischia: most inferior point |
| 17-18 | Pubis: most anterior midpoint |
| 19-20 | Acetabulum: most inferior-anterior point on the ischium part of the lunate surface |
| 21-22 | Acetabulum: deepest point on acetabular fossa |
| **23-24** | Acetabulum: most inferior-anterior point on the pubic part of the lunate surface |
| **25-26** | Acetabulum: most superior-anterior point on the ischium part of the lunate surface |
| **27-28** | Iliopubic juncture: anterior fusion point on the pubic bone |
| **29-30** | Iliopubic juncture: anterior fusion point on the ilium bone |
| 31-32 | Ilium: most posterior point below the Spina iliaca |
| 33-34 | Spina iliaca: most anterior-inferior point |
| 35-36 | Ilium: most posterior point between the spina iliaca anterior-inferior and anterior-superior points |
| 37-38 | Spina iliaca: most anterior-superior point |
| 39-40 | Crista iliaca: most superior point |
| 41-42 | Spina iliaca: posterior-superior point |
| 43-44 | Spina iliaca: posterior-inferior point |
| 45-46 | Incisura ischiadica major: deepest point |
| **47-48** | Ilioischial juncture: ilium-acetabulum fusion point |
| **49-50** | Ilioischial juncture: ischium-acetabulum fusion point |
| 51-52 | Ilioischial juncture: ilium fusion midpoint |
| 53-54 | Ilioischial juncture: ilium lateral fusion point |
| 55-56 | Ischium: most anterior point above the Corpus ossis ischii |
| 57 | S1: most superior point on S1 center |
| 58 | S1: most superior-anterior point |
| 59 | S1: most anterior midpoint |
| 60 | S2: most anterior midpoint |
| 61 | S3: most anterior midpoint |

Bold indicates LM that fuse in adults and for which the mean position was calculated

| 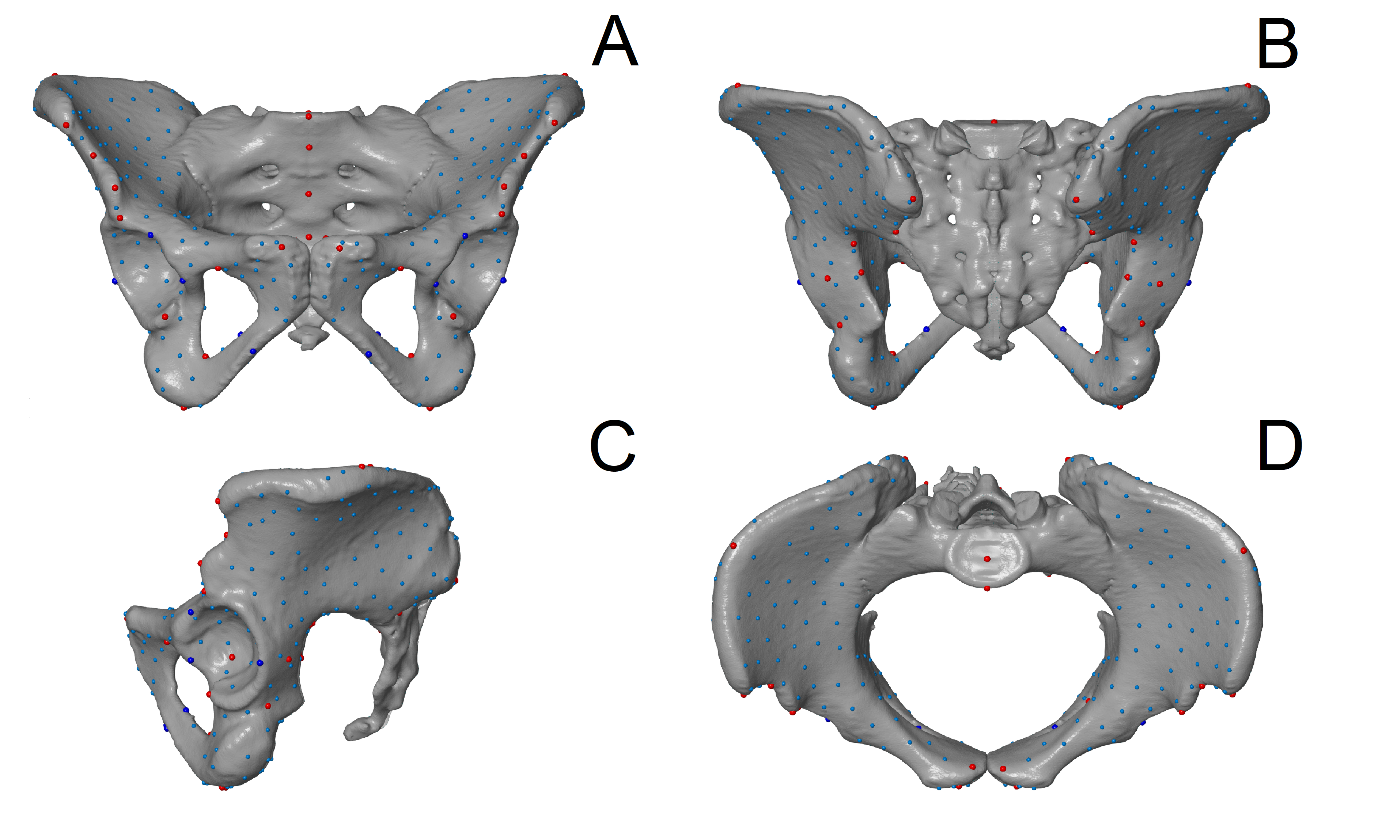 |
| --- |
| Fig. 1S. Location of the 61 LMs on a mature pelvic girdle. Red dot, surface LM; dark blue dot, LM fused in adults; light blue dot, SLM |
| 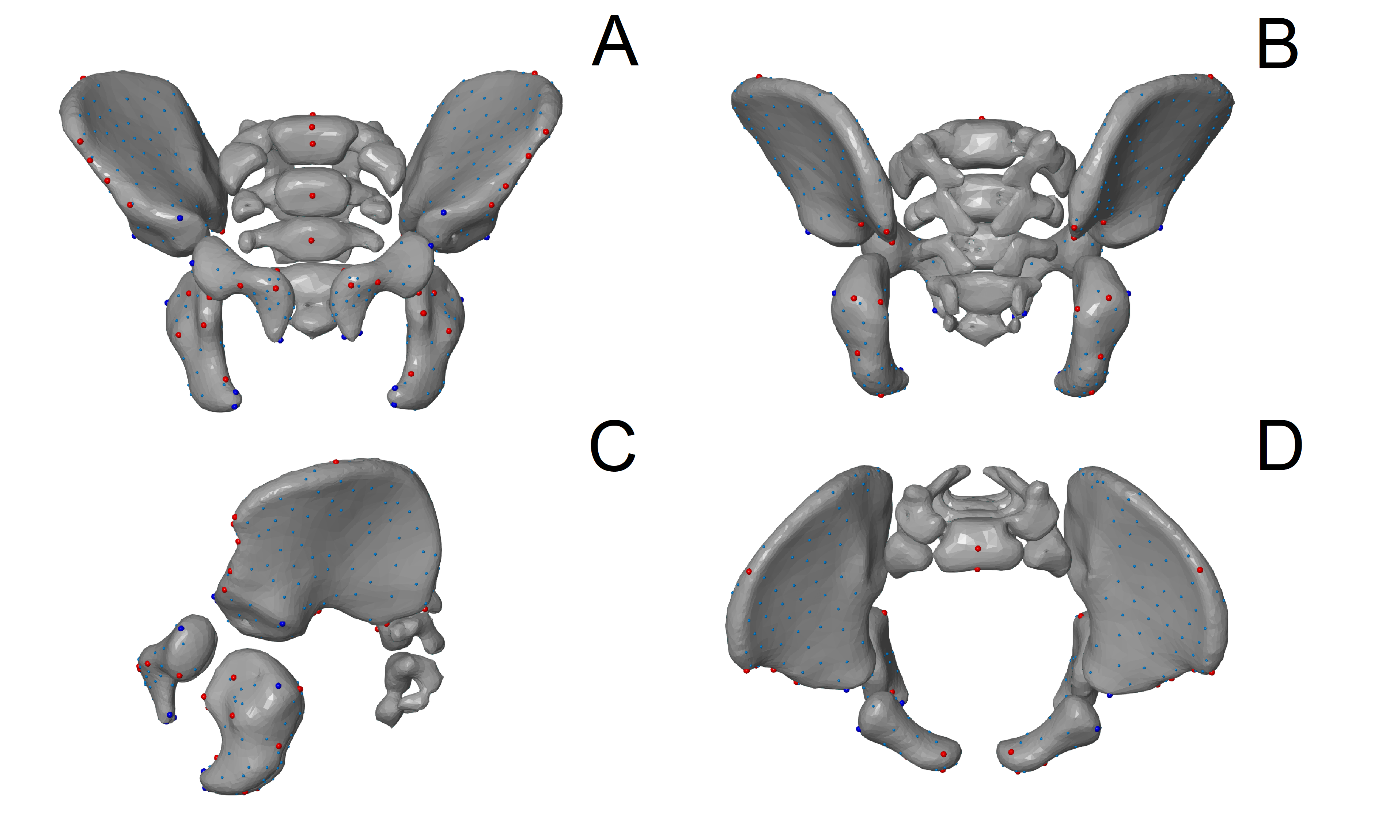 |
| Fig. 2S. Location of the 61 LMs on an immature pelvic girdle. Red dot, surface LM; dark blue dot, LM fused in adults; light blue dot, SLM |

The first 61 landmarks (LM) were digitized on one 3D pelvic reconstruction chosen as a template. Next, 476 surface semilandmarks (SLM) were automatically generated from this template mesh using the 3D Slicer software. The pelvic mesh template with 476 SLM was warped to all remaining pelvic girdles based on the location of the 61 LM of each individual (Table 3S). Next, the warped 476 SLM were slid along the tangents using the SlicerMorph extension in 3D Slicer until they contacted the mesh surface and their total bending energy of a thin plate spline (TPS) was minimized ^4^. All 61 LM and 476 SLM (i.e., raw coordinates) were superimposed using a Generalized Procrustes Analysis (GPA). GPA consists of a three-step process (i.e., translation, rotation, and standardization) which removes the differences in location, orientation, and scale of LM and SLM between meshes and leaves only differences in shapes ^5^. The result of the GPA was the Procrustes coordinates and a size variable called the centroid size (i.e., the square root of the sum of squared distances between the centroid and all other landmarks)^5,6^. Since the LM and SLM were digitized by one observer, an intra-observer error was calculated for 30 randomly selected pelvises using a Procrustes ANOVA. The magnitude of error was derived from the Procrustes ANOVA’s mean squares (MS), that shows how much more variation is at the particular level than at the lower level. The mean squares of the measurement error (i.e., double distribution of LM and SLM; MS=9.8×10^-6^) was 18 times smaller than the individual variation (MS=1.8×10^-4^); therefore, the digitizing error was acceptable small relative to the biological variation in pelvic shape.

The next step was to assess how much of the shape variation is explained by pelvic size. Therefore, multivariate regression with a permutation test was performed where shape (i.e., Procrustes coordinates) was the dependent variable and pelvic size (i.e., centroid size) was the independent variable. The results were statistically significant (Table 4S) in both young and old groups. Ontogenetic allometry explained a large fraction of total shape variation, especially in the young group (30%). Therefore, all further geometric morphometric analysis was performed on the regression residuals to control for ontogenetic allometry^7^. This approach assures that the obtained results are the effect of the variation in geometric shape and are not biased by the pelvic size (especially in the young group, which is characterized by high variation in the centroid size).

Table 4S. Multivariate regressions between Procrustes coordinates and centroid size in the young and old groups.

| Sample | N | Predicted [%] | *P* |
| --- | --- | --- | --- |
| Young group (<25) | 100 | 30.00 | **<0.0001** |
| Old group (>25) | 208 | 1.79 | **0.0465** |

N – number of individuals in the group, Predicted % - percent of the predicted shape, P – p-value of the permutation test with 10000 randomization rounds, bold indicates statistically significant p-value at level <0.05.

Table 5S. Detailed information on the multivariate regression between pelvic shape and the BMI categories in the young and old groups by sex

| Sample | Female | | | | | | Male | | | | | |
| --- | --- | --- | --- | --- | --- | --- | --- | --- | --- | --- | --- | --- |
|  | N | Total SS | Pred. SS | Resid. SS | Predicted [%] | *P* | N | Total SS | Pred. SS | Resid. SS | Predicted [%] | *P* |
| Young group (<25) | 50 | 0.168 | 0.009 | 0.159 | 5.48% | **0.008** | 50 | 0.131 | 0.003 | 0.128 | 2.8% | 0.166 |
| Old group (>25) | 107 | 0.307 | 0.003 | 0.304 | 1.28% | 0.178 | 101 | 0.270 | 0.007 | 0.263 | 2.61% | **0.004** |

N – number of individuals in the group, Total SS – a total of sums of squares, Predicted SS – predicted sums of squares, Residuals SS – residuals of sums of squares, Predicted % - percent of the predicted shape, P – p-value of the permutation test with 10000 randomization rounds, bold indicates statistically significant p-value at level <0.05

Table 6S. Sample size by the number of live births in the females aged 25-45

| Live births | N |
| --- | --- |
| 0 | 10 |
| 1 | 17 |
| 2 | 6 |
| 3 | 8 |
| 4 | 1 |
| 5 | 0 |
| 6 | 1 |

N - number of females

**Results**

Principal Component Analysis was performed on the multivariate residuals and the complete data set. The first principal component (PC1) separates the pelvic shape between females and males (Fig. 1). PC1 (27.35%) represents ilium bending and widening and subpubic angle size. PC2 (17.26%) explains the width of the pelvic inlet and the bending of the ischium (Fig. 1).

| 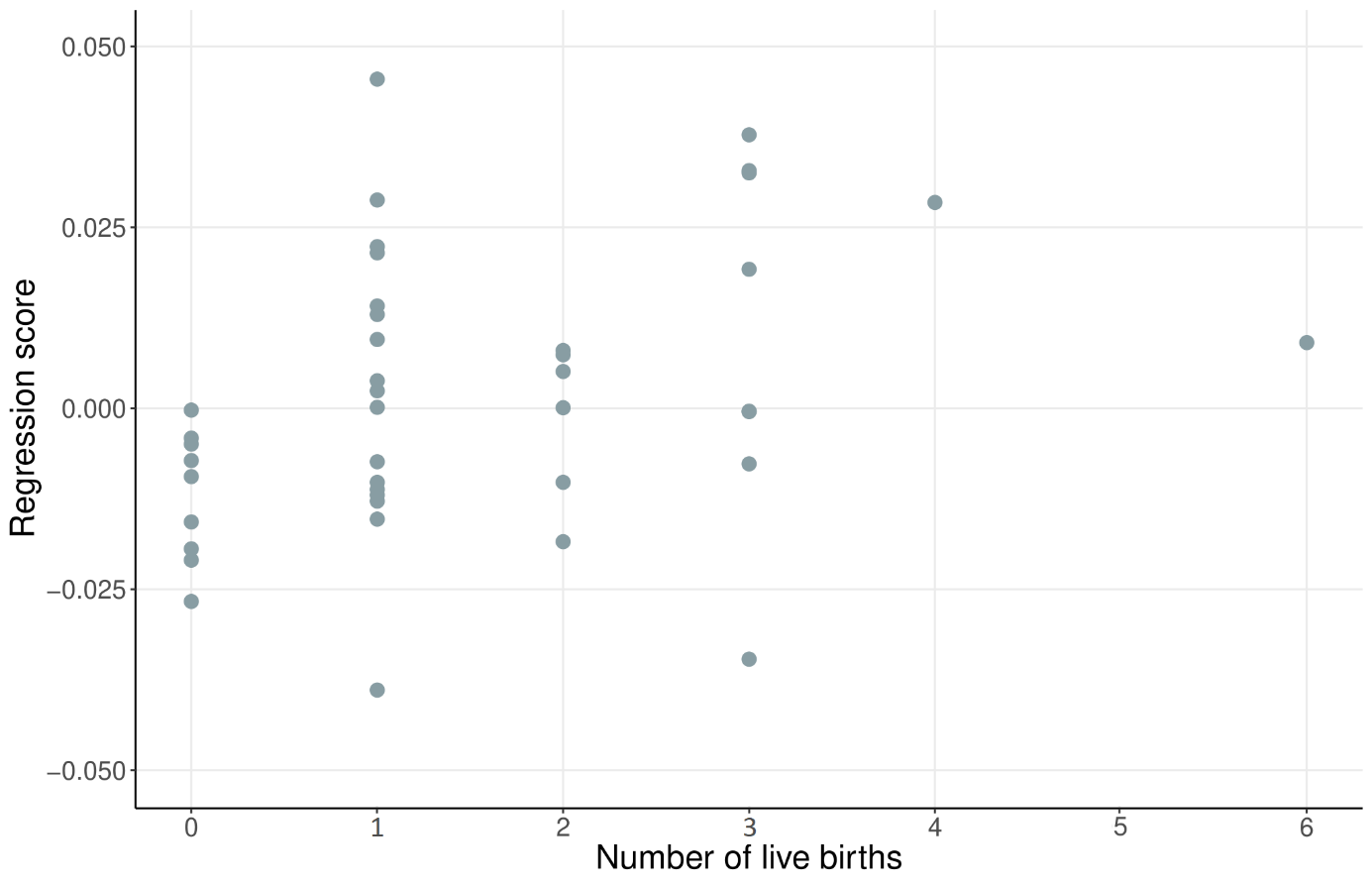 |
| --- |
| Fig. 3S. The relationship between the number of live births and pelvic shape (Procrustes coordinates) in females aged 24–45 |

**Movie legend**

Movie 1S. Changes in pelvic shape along the PC1 axis in the anterior-posterior plane

Movie 2S. Changes in pelvic shape along the PC1 axis in the superior-inferior plane

Movie 3S. Changes in pelvic shape along the PC2 axis in the anterior-posterior plane

Movie 4S. Changes in pelvic shape along the PC2 axis in the superior-inferior plane

Movie 5S. Changes in pelvic shape in young females from underweight to obese BMI in the anterior-posterior plane

Movie 6S. Changes in pelvic shape in young females from underweight to obese BMI in the superior-inferior plane

Movie 7S. Changes in pelvic shape in young females from underweight to obese BMI in the medio-lateral plane

Movie 8S. Changes in pelvic shape in old males from underweight to obese BMI in the anterior-posterior plane

Movie 9S. Changes in pelvic shape in old males from underweight to obese BMI in the superior-inferior plane

Movie 10S. Changes in pelvic shape in old males from underweight to obese BMI in the medio-lateral plane

**References**

1. Edgar, H. *et al.* New Mexico Decedent Image Database. *Office of the Medical Investigator, University of New Mexico* (2020) doi:doi.org/10.25827/5s8c-n515.

2. Musielak, B. *et al.* Variation in pelvic shape and size in Eastern European males: A computed tomography comparative study. *PeerJ* **2019**, (2019).

3. Musielak, B. J. *et al.* Is acetabular dysplasia and pelvic deformity properly interpreted in patients with congenital femoral deficiency? A 3D analysis of pelvic computed tomography. *J. Child. Orthop.* **14**, (2020).

4. Bardua, C., Felice, R. N., Watanabe, A., Fabre, A. C. & Goswami, A. A practical guide to sliding and surface semilandmarks in morphometric analyses. *Integr. Org. Biol.* **1**, 1–34 (2019).

5. Zelditch, M. L., Świderski, D. L., Sheets, H. D. & Fink, W. L. *Geometric Morphometrics for Biologists: A Primer*. (Elsevier Academic Press, 2004).

6. Cooke, S. B. & Terhune, C. E. Form, Function, and Geometric Morphometrics. *Anat. Rec.* **298**, 5–28 (2015).

7. Outomuro, D. & Johansson, F. A potential pitfall in studies of biological shape: Does size matter? *J. Anim. Ecol.* **86**, 1447–1457 (2017).
